# Lay testing cadres and point-of-care diagnostic tests for HIV and other diseases: An essential combination in health service delivery

**DOI:** 10.1371/journal.pmed.1003867

**Published:** 2021-11-24

**Authors:** Zibusiso Ndlovu, Tom Ellman

**Affiliations:** Médecins Sans Frontières, Southern African Medical Unit, Cape Town, South Africa

## Abstract

Zibusiso Ndlovu and Tom Ellman discuss the potential value of task sharing in provision of testing for HIV and other infectious diseases.

**Summary points**

- Lack of access to testing plays a major role in the underdiagnosis of infectious and noncommunicable diseases, leading to higher morbidity and mortality.
- Point-of-care (POC) tests can offer rapid results, allowing for timely initiation of therapy. However, mere availability of POC tests in health facilities does not ensure utilization. Conducting POC tests has been shown to be a burden on highly trained frontline healthcare workers (HCWs; clinicians and nurses), who often have a broader scope of responsibilities and are critically scarce in many settings.
- The continual emergence of easy-to-use POC tests has not been accompanied by investment in a cadre of health workers to support their delivery, especially at decentralized health facilities where patients initially seek healthcare support.
- Historically, implementation of task shifting for POC tests has proven difficult due to lack of integration into national human resource structures and fiscal plans and lack of explicit national policies promoting task shifting, together with resistance from laboratory professionals.
- We propose that the scope of work for existing lay health worker (LHW) cadres could be broadened/remodeled to include POC tests for HIV services including for advanced HIV disease and other priority diseases, especially in primary healthcare or lower level facilities without laboratories. Policy makers and national program managers could ensure that this is part of broader national health workforce policies.
- Concerns of professional and/or regulatory bodies must be addressed, and these bodies (medical and laboratory councils) can guide national policy on which POC tests can be task shifted to less laboratory-trained cadres, and they could also lead in the development of a framework of education and supervision to ensure sustainability and maintenance of professional standards.
- Lay testing initiatives can scale up access to the multitude of available essential POC tests, for maximal impact of disease testing, closer to where people live. This can improve global health and accelerate progress toward universal health coverage.

## Introduction

Point-of-care (POC) diagnostic testing is widely recognized as an essential component of ensuring access to health services [1,2], reducing delays in the diagnosis, prompt treatment initiation, and facilitating linkage to care [1–3]. POC testing has been crucial in enabling access to Antiretroviral Therapy (ART) and to the decentralization of HIV services, including task sharing of diagnostics with lay health workers (LHWs) and patient self-testing.

An array of simple and relatively affordable POC tests are available and can help to identify advanced HIV disease and other communicable (tuberculosis, malaria, syphilis, and hepatitis) and noncommunicable diseases (diabetes). Yet, these tests are underused, and in many settings, they remain centralized or dependent on slow and lengthy referral of samples and result delivery. In primary healthcare facilities in resource-limited settings, POC testing can be the only viable route when centralized testing is hours away.

Low testing rates and underdiagnosis for communicable and noncommunicable diseases among key populations is commonly reported [4–6]. Mere availability of POC tests does not ensure their adoption and utilization. Conducting POC test has been shown to be a burden on highly trained frontline healthcare workers (HCWs; clinicians and nurses) [3], and in many settings, chronic critical shortage of these HCWs (who often have a broader scope of responsibilities), coupled with other health system challenges, hampers expansion of essential POC diagnostic services [3,7]. Laboratory-trained technicians are also scarce especially in rural settings and often not present in primary healthcare facilities.

A commission on diagnostics concluded that 47% of the world’s population have little or no access to diagnostics and that the diagnostic gap is most severe at primary healthcare level where only about 19% of populations in low- and middle-income countries have access to the simplest diagnostic tests (other than for HIV and malaria) [4].

The world is offtrack to end AIDS in 2030. An estimated 1.5 million people became newly infected with HIV in 2020, a figure 3 times higher than the global Joint United Nations Programme on HIV/AIDS (UNAIDS) target of less than 500,000 new infections by the year 2020, and nearly 700,000 people lost their lives to AIDS-related illnesses [8]. The Coronavirus Disease 2019 (COVID-19) pandemic has had a devastating impact on the continuity of HIV services [9]. Equally, control and elimination targets for many other multiple priority diseases that cause significant health burden, including tuberculosis, noncommunicable diseases, sexually transmitted infections, malaria, neglected tropical diseases, and hepatitis [10–14] among others, are affected.

Since 2008, WHO has recommended task sharing (rational redistribution of tasks from higher-level cadres of health professionals to trained LHWs) within the HIV continuum of diagnosis, prevention, care, and treatment [15], and in the 2021 HIV service delivery guidelines, WHO strongly recommended task sharing of specimen collection and POC testing with nonlaboratory personnel when professional staffing capacity is limited [16]. LHWs are defined as “any health worker who performs functions related to health-care delivery, trained in some way in the context of the intervention; but with no formal professional or paraprofessional certificate or tertiary education degree” [15].

We argue that a course correction is needed, and it involves strengthening and formalizing LHWs for sample collection and POC testing, especially at primary healthcare sites, for HIV services including for advanced HIV disease and other priority diseases with significant unmet testing needs. We also argue that careful consideration is needed in each context for the mix of laboratory and other tasks to be shared according to priority needs, workflows, and care processes, especially in low- and middle-income countries.

The continual emergence of simplified POC tests has not been associated with an accompanying remodeling of human resources at health facilities for maximal impact of disease testing. At POC level, performing POC testing is usually conducted by frontline HCWs who are frequently rotated through the system, and as the repertoire of POC tests increases, their usage may inevitably diminish. Primary healthcare facilities are predominantly the first point for seeking healthcare among patients with acute and chronic conditions and can enable earlier detection of diseases before patients develop severe conditions that necessitate hospital admission. Use of simple POC tests at decentralized facilities by LHWs can mitigate the lack of laboratory staff and reduce work overload among scarce HCWs.

### Task sharing in healthcare services

LHWs have been used increasingly, especially in the context of disease prevention, health promotion, diagnostic services, treatment support, counseling, and home-based care. Reviews have shown that they are referred to using many different titles, mostly depending on their roles (expert clients, lay counselors, community health workers, health diagnostic assistants, health surveillance assistants, and mentor mothers) [17–21].

Studies have shown that LHWs can significantly expand uptake of HIV testing services at health facility and community and outreach levels, including among key populations [22–25]. Studies and program experiences have also shown that LHWs can provide high-quality POC testing for CD4 cell count, cryptococcal antigen, urinary mycobacterial tuberculosis lipoarabinomannan antigen, malaria, syphilis, and pregnancy, including near POC molecular testing for HIV early infant diagnosis, HIV viral load, and tuberculosis through Cepheid GeneXpert system [1,3,26–28]. However, other studies have found increased error rates from the Cepheid GeneXpert MTB/RIF and Alere q Detect, when operated by nonlaboratory staff [29,30].

Nonetheless, task-shared POC testing has been shown to have time-saving benefits for HCWs, improve testing coverage and provide prompt results for expedited patient management [31–33], among other benefits. A study of national policies for HIV testing services across 50 countries showed that only 42% allowed LHWs to perform testing using POC tests (64% in African countries), although more allowed LHWs to perform pre- and posttest counseling (56% overall and 80% in Africa) [34]. The study also revealed that several countries explicitly limit these roles to trained healthcare providers due to concerns about LHWs’ ability to perform POC tests [34]. The study highlighted that 13/50 (26%) of the reviewed country policies did not explicitly outline the role of LHWs in providing HIV testing services, and this could introduce barriers to task shifting. In-country national program management should lead on efforts to accelerate national policy reviews to include explicit reference to the role of LHWs in HIV testing services.

## Case studies: Task sharing experiences for POC testing

In 2015, Malawi, with the support of nongovernmental organizations (NGOs), deployed over 1,100 LHWs (health diagnostic assistants) to health facilities across the country to conduct HIV and syphilis POC testing among other roles. At least 2.6 million patients tested for HIV were attributable to the health diagnostic assistant intervention [31]. However, interventions (such as this in Malawi), which are excessively reliant on donor funding and not wholly integrated into national structures, remain fragile and unsustainable. The impact of terminating task shifting services for POC testing among LHWs has been shown in South Africa, where a redeployment policy for HIV lay counselors in 2015 led to fewer HIV tests carried out in clinics [35].

In a largely rural and extremely poor district of Malawi (Nsanje), ministry of health and NGOs successfully deployed health diagnostic assistants to primary healthcare facilities and a district hospital to conduct advanced HIV disease testing (CD4 cell count, urine mycobacterial tuberculosis lipoarabinomannan antigen, and cryptococcal antigen), and over 920 patients were diagnosed with advanced HIV disease within a year compared to before when there were neither POC diagnostics nor efficient conventional referral options for advanced HIV disease testing [36]. Identifying and replicating practices with greater evidence of effectiveness are increasingly important, as countries and partners work together within a shrinking fiscal landscape.

## Challenges to the implementation of task shifting for POC testing

Implementation of task shifting for POC testing has proven difficult in practice, and support for lay testers and their integration into health systems within countries has been uneven and mostly driven by NGOs, without any longer-term perspective [31,36]. In many settings, lack of integration of task shifting into national human resource structures and fiscal plans, together with lack of explicit national policy or strategic plans promoting task shifting for POC testing, has also aggravated barriers to task shifting [37]. Legal structures, which enforce strict professional boundaries, have historically limited possible extension of the task shifting scope for the multitude of POC tests. The perceived need for the protection of professional turf by other health cadres, especially laboratory technicians and/or their professional or regulatory bodies not willing to endorse tasked-shifted POC/near POC testing, as seen in some settings [38,39], including monopolistic elements from the diagnostic fraternity, has also contributed to barriers to adoption of task shifting. Other barriers for task shifting for POC testing include general reluctance for adoption within healthcare centers (institutional resistance) together with incoherent existing programs aiming to support task shifting, for example, implementation of reflex urine mycobacterial tuberculosis lipoarabinomannan antigen and cryptococcal antigen testing at primary healthcare level yet within a system of centralized laboratory CD4 testing [40].

## Recommendations for a lay testing implementation framework

### Sources of LHWs

Rather than deploying new LHWs for this specific role, we propose that national program managers should review roles and responsibilities between the various existing LHW profiles together with an analysis of the diagnostic POC testing network (workloads, gaps in testing, testing flow, quality of POC testing services, and responsibilities of testing), especially at primary healthcare level. National health programs presently have different LHWs (and/or HCWs) within the human resource structure (Table 1), who could be offered extra user training and could potentially take up this role for lay testing for diagnostic services. For example, as intensive HIV pretest counseling (with individualized risk assessment) is no longer recommended because it may create barriers to service delivery [41,42], and as other HCWs are now equally capable of providing this pretest information and or even conduct HIV testing, lay providers primarily specific for HIV testing services could possibly have their scope broadened to include conducting other POC diagnostic tests. In many settings, LHWs have a minimum education level of a high school leaving certificate [31] and where needed, primary healthcare level or district managers can liaise with community leaders to identify eligible school-leavers to support this role.

**Table 1:** Panel with list of potential lay testers, the POC tests they could conduct, and other activities.

Lay and/or professional health workers in programs	POC tests that can be task shifted, including other activities	Test-related tasks for LHWs
• HIV testing services counselors • Health diagnostic assistants • CHWs • Health facility navigators • Lay counselors • Nurse assistants • Phlebotomists • Laboratory assistants • Microscopists • A new cadre	HIV rapid diagnostic tests, basic advanced HIV disease^**§**^ testing package (CD4 cell count, cryptococcal antigen, and urinary mycobacterial tuberculosis lipoarabinomannan antigen test), syphilis rapid diagnostic test, pregnancy test, hemoglobin A1c meters, urinalysis test strips, glucometers, SARS-CoV-2 rapid diagnostic test, *Vibrio cholerae* antigen test, hepatitis B and C rapid diagnostic test, malaria rapid diagnostic test, haemoglobinometer, POC nucleic acid tests for HIV viral load, HIV early infant diagnosis, and MTB/Rif	Navigate priority results, QC for all POC tests, referrals of samples to higher tier laboratories (hubs) and follow-up on results, stock management of test devices, and ancillary reagents
	Sample collections: Dry blood spot for HIV viral load and early infant diagnosis of HIV, urine, sputum, nasopharyngeal, finger prick, and venous blood	

^**§**^Advanced HIV disease is defined as an adult, adolescent, or child greater than 5 years old with a CD4+ cell count less than 200 cells/mm^3^ or a WHO clinical stage 3 or 4 event as well as all children less than 5 years of age [16].

CHW, community healthcare worker; LHW, lay health worker; POC, point-of-care; QC, quality control; SARS-CoV-2, Severe Acute Respiratory Syndrome Coronavirus 2.

### Policy needs

Although there is growing awareness of WHO guidelines for managing advanced HIV disease in many countries, rollout remains slow, and explicit policies for promoting lay testing for advanced HIV disease services are also lacking. We propose development of policies for supporting lay testing to help scale up advanced HIV disease and other HIV-related services. Concerns of professional and or regulatory bodies and individual practitioner prejudices must be firmly addressed for policy to be agreed and implemented. These concerns could be related to professional protectionism, financial interests, and/or pertain to anticipated reduced quality of task-shifted POC testing activities, among other reasons. A broader engagement of all stakeholders, including sharing of evidence, could help to explicitly address these concerns. Regulatory bodies (medical and laboratory councils) hold the crucial position of potentially guiding national policy on which POC tests can be task shifted to less laboratory-trained cadres. With the support of national program managers, regulatory bodies could also lead in developing the framework for LHW POC test user training, certification, and ongoing supervision, especially for deployment in PHC or lower level facilities without laboratories. Regulatory bodies also have the influence to quell any resistance to task shifting from their members.

### Training and remuneration

Critical to the success of LHW testing programs will be the provision of standardized and coordinated accredited diagnostic testing training (initial and for new content) with competency-based formal certification [43]. Accredited training can increase confidence and job satisfaction and lead to rapid career progression, including the formal recognition of LHWs within health facilities. Even though the pedagogy and content of training programs may be different, they should cover context/region and national specific disease priorities, which could include HIV/AIDS, sexually transmitted infections (STIs), malaria, tuberculosis (TB), and noncommunicable diseases, among others. LHWs should receive fair and consistent wages commensurate with workload and national standards, and governments should be responsible for regulating wages to ensure uniform payment across programs [43]. LHWs represent a cadre who require less training, education, and salary and are therefore more likely to be found within rural communities where their knowledge of the community and low level of mobility will further add value to the role.

### Polyvalency of LHWs

LHWs should not be narrowly disease focused in their POC testing activities but should prioritize tests and testing-related tasks according to the major health needs among the population and the balance of needs and gaps within the workflow of the facility. They could be responsible for a variety of diagnostic tests including POC tests for HIV, together with other sexually transmitted infections, advanced HIV disease tests (CD4 cell count, urine mycobacterial tuberculosis lipoarabinomannan antigen, and cryptococcal antigen), plus other POC/near POC tests listed in Table 1.

### Maintaining quality POC testing services and sustainability

Task shifting should not compromise the quality of diagnostic testing and professional standards, and it should happen within legal protection for those with appropriate levels of competency, together with protecting patients. Indicators of monitoring an acceptable level of quality of testing must be developed, and these can include daily/weekly internal controls, routine blinded external quality controls, and ongoing supportive supervision from hub laboratories including auditing and accreditation of testing facilities (physical space for testing, storage place for test kits and stock management, testing algorithm used, job aides for testing, documentation in testing logbook, and safety aspects). Because there are possibly existing LHWs whose scope could be broadened/remodeled to include lay testing in many settings, long-term sustainability of this framework could be assured as long as there is a conducive environment for processes, which may improve the quality of service delivery (Table 2). We encourage national program managers to conduct promotional activities to increase awareness, demand, and mainstream utilization/deployment of lay testers for providing HIV testing services including for advanced HIV disease and other priority diseases.

**Table 2:** Health system enablers for implementing lay testing.

Health system enablers for lay testing
• Ensure explicit policies on task shifting and clarity on the range of POC tests that can be performed by LHWs • Ensure the availability of POC tests, including at primary care level as recommended in WHO essential diagnostics list [47] (or national essential diagnostic list) • Formalize accredited training with certification for lay testers including refresher training and/or for other newer POC technologies • Set up framework for ongoing supervision, technical support, and QA systems for lay testing services • Consider creating an enabling environment for LHWs to belong to a health professional body for ongoing capacity development • Strengthen mechanisms to ensure that LHW testing results are used for patient management • Trained providers who have not practiced for more than a year should undergo refresher trainings before they resume POC testing

LHW, lay health worker; POC, point-of-care; QA, quality assurance.

Although diagnostic technology and LHWs are part of the solution for scaling up diagnostic testing services, this is only effective when implemented within functioning health systems where results are used for patient management with availability of therapeutic commodities.

The need for lay testing at district, regional, provincial or specialized hospitals, and national reference laboratories is less paramount as they usually have trained laboratory technologists/specialists. However, for POC testing at outpatient, inpatient, and even at emergency department in hospitals, lay testing could be considered especially where laboratory-based result turnaround time preclude clinical utility.

International funding organizations should also encourage countries to prioritize lay testing for HIV, advanced HIV disease, and other priority diseases.

## Cost-effectiveness of LHW deployment

Lay testing is generally considered to be less expensive compared with other cadres of health workers, especially with regard to remuneration and incentives as well as training and supervision costs [31]. However, costing and cost-effectiveness of lay testers compared to standard of care has not been well documented, and studies are required to provide persuasive health economic arguments to policy makers.

## Other opportunities

While this viewpoint focused mostly on task shifting for HIV-related POC tests, the same lay testing implementation framework will likely apply to other priority disease POC tests. The role of lay testers should be considered in a long-term perspective as their tasks may evolve over time in parallel with changes in the diagnostic pipeline or disease outbreaks and other health system requirements. For example, LHWs would be crucial in conducting COVID-19 antigen rapid diagnostic tests at POC to aid in the diagnosis of COVID-19, particularly in settings where nucleic acid testing turnaround times preclude clinical utility. LHWs may eventually perform other essential POC tests within the long pipeline including multiplex POC tests for infectious diseases like HIV/syphilis dual testing, POC urine–based tenofovir, semiquantitative cryptococcal antigen, and some requiring significant hands-on time (Visitect CD4 lateral flow assay, urine FujiLAM), among other tests. Programs should have sufficient investments in lay testing that could ensure adequate utilization of these tests.

Since 2018, WHO has published a yearly essential diagnostics list that details recommended in vitro diagnostics that should be available at community level, primary care, and in all laboratories [44]. For tests conducted outside laboratory settings, the essential diagnostics list should highlight the tester’s skills required to conduct the test (professionally trained or lay cadre trained) together with the level of POC test ease of use. Such language from WHO could assist countries to further develop national policies on the extent of the acceptable range of POC tests that can be conducted by less-trained health workers especially at primary healthcare level including health posts, community settings, outreach clinics, and ambulatory care settings, including among key population groups.

## Further research agenda

Observational and modeling studies to identify the best mix of tasks and allocation of LHWs including comparison with alternative possible approaches according to the burden and flow in specific contexts are needed. Further studies on performance and quality of LHW POC testing services, cost-effectiveness, and determination of supervision needs at primary healthcare level to help ensure improved health outcomes are encouraged.

Promotion of lay testing cadres should synergize with efforts for development of POC tests for other diseases, which could include genital chlamydial and gonococcal infections, blood level ART adherence, active TB biomarkers, and common causes of fevers including among patients with comorbid conditions, among other disease POC tests.

## Conclusions

POC lay testing is an area of high priority for investment in primary healthcare facilities and can enable equitable access to the multitude of essential POC diagnostics. As new tests and treatments potentially enable further decentralization of essential healthcare to remote communities, the long turnaround times or delays in access associated with centralized services will become increasingly unacceptable. Lay testing cadres, facilitating access to these tools, will become a critical part of a strengthened health system and a remodeled workforce, able to support decentralization of quality care.

## Abbreviations

ART: Antiretroviral Therapy
COVID-19: Coronavirus Disease 2019
HCW: healthcare worker
LHW: lay health worker
NGO: nongovernmental organization
POC: point-of-care
STI: sexually transmitted infection
TB: tuberculosis
UNIAIDS: Joint United Nations Programme on HIV/AIDS
